# Analysis of gut microbiota and fecal/plasma short-chain fatty acids in non-dialysis renal anemia patients: associations with clinical parameters

**DOI:** 10.3389/fmicb.2026.1838946

**Published:** 2026-09-07

**Authors:** Lin Yang, Lifen Liu, Pingping Kong, Wenwen Xu, Zhiyuan Wang, Siqi He, Jing Lou, Yanna Dou

**Affiliations:** 1 Pediatric Surgery Department, The First Affiliated Hospital of Zhengzhou University, Zhengzhou, China; 2 Nephrology Department, The First Affiliated Hospital of Zhengzhou University, Zhengzhou, China

**Keywords:** chronic kidney disease, gut microbiota, non-dialysis, renal anemia, short-chain fatty acids (SCFAs)

## Abstract

Renal anemia is one of the most common complications of chronic kidney disease (CKD). This study investigated gut microbiota composition and fecal/plasma short-chain fatty acids (SCFAs) in 30 non-dialysis renal anemia patients and 20 healthy controls. Gut microbiota was assessed by 16S rRNA gene amplification sequencing and SCFAs were measured using gas chromatography-tandem mass spectrometry (GC–MS/MS). Compared with healthy controls, renal anemia patients showed distinct gut microbiota composition, including higher relative abundances of Enterobacterales, Enterobacteriaceae, Escherichia-Shigella, and *Escherichia coli*, and lower abundance of Lachnospiraceae. Fecal acetic acid was higher in renal anemia patients than in controls (*p* = 0.049). Plasma acetic acid was also increased in renal anemia patients (*p* = 0.027), whereas plasma isobutyric acid (*p* = 0.002) and caproic acid (*p* = 0.035) were higher in controls. In subgroup analysis, moderately anemic patients had higher fecal isobutyric acid (*p* = 0.019) and isovaleric acid (*p* = 0.028) than mildly anemic patients. Specific microbial taxa and SCFAs were correlated with clinical indicators of renal anemia. These findings suggest that renal anemia is associated with gut microbiota dysbiosis and alterations in specific SCFAs in the context of CKD, but further studies with eGFR-matched non-anemic CKD controls are needed to clarify the independent contribution of anemia.

## 1 Introduction

Renal anemia is one of the most common complications of Chronic Kidney Disease (CKD), which is estimated to influence 2 to 4 million of the approximately 20 million people in the United States with CKD (Dowling, 2007). A cross-sectional study of anemia in Chinese patients with non-dialysis CKD also revealed that renal anemia has a high prevalence, a high incidence, and a low awareness in China (Li et al., 2016). Emerging evidence indicated that renal anemia may lead to neurocognitive impairment, sleep disturbances, CKD progression, cardiovascular comorbidities, higher hospitalization rates, and mortality, which impose a heavy physiological and economic burden on patients and a disastrous effect on public health (Schlender et al., 2021; Huang et al., 2022; Lv et al., 2022; Smith, 2010; van Nooten et al., 2010). According to KDIGO guidance, the evaluation and management of renal anemia remain important components of contemporary CKD care (Kidney Disease: Improving Global Outcomes (KDIGO) CKD Work Group, 2024). Unfortunately, the prevalence of renal anemia is still continuously increasing worldwide. Renal anemia is a multifactorial process caused by erythropoietin (EPO) deficiency, decreased responsiveness to EPO, shortened erythrocyte survival, disordered iron homeostasis, and chronic inflammation (Babitt and Lin, 2012). Corresponding with these factors, the treatment strategies for anemia in CKD mainly include the use of erythropoiesis-stimulating agents (ESAs), iron therapy and hypoxia-inducible factor-prolyl hydroxylase inhibitors (HIF-PHIs), which has made significant process in recent years (Bonomini et al., 2016). Nevertheless, each of them has been found to have some limitations. For example, previous studies found that iron supplementation is unsafe or even deadly in countries with poor medical care, the ESAs may increase the incidence of cardiovascular events and vascular access thrombosis, and long-term use of HIF-PHIs may exert potential effects on tumor growth (Gupta and Wish, 2017; Jonker and Boele van Hensbroek, 2014). Moreover, research revealed that treatment of anemia in the predialysis and dialysis populations remains suboptimal (Li et al., 2016). Therefore, it is necessary to explore the potential pathogenesis and novel therapeutic approach of renal anemia.

In recent years, gut microbiota has been extensively studied, which plays a vital role in human intestinal homeostasis and is associated closely with the progression of numerous diseases (Thursby and Juge, 2017). Emerging evidence indicates that gut dysbiosis may be implicated in the pathogenesis of CKD with underlying mechanisms that may include metabolic disorder, immune disorder, and neuroendocrine disorders (Yang et al., 2018; Meijers et al., 2019). The interaction between the intestine and the kidney is known as the gut-kidney axis. The gut-kidney axis hypothesis has led to numerous studies showing that patients with CKD have a distinct gut microbiota structure compared with controls and that changes correlate with disease severity and progression (Lehto and Groop, 2018). The study reported that targeting metabolites produced by *Lactobacillus johnsonii* can reverse CKD (Miao et al., 2024a) and *Lactobacillus* can ameliorate CKD through regulating gut microbiota-derived indole metabolites (Miao et al., 2024b). The short-chain fatty acids (SCFAs) are the main metabolites produced by dietary fibers and resistant starch in the presence of gut microbiota, which plays a vital role in microbiota homeostasis and is speculated to be pivotal in the gut-kidney crosstalk (Briskey et al., 2017; den Besten et al., 2013). Short-chain fatty acids are absorbed in the gastrointestinal tract and carried to the liver through the portal vein, with some also entering the systemic blood circulation. Acetic acid, propionic acid, and butyric acid are the most abundant SCFAs in the colon (Cummings et al., 1987). SCFAs have been demonstrated to reduce inflammatory responses in kidney ischemia–reperfusion injury (Andrade-Oliveira et al., 2015)and delay the progression of diabetic nephrology (Li et al., 2020). The SCFAs can also affect the CKD patients by attenuating the inflammatory response, inhibiting oxidative stress, modulating autophagy, and regulating energy metabolism and immune pathways (He et al., 2024; Zheng et al., 2023; Zhou et al., 2024; Ye et al., 2024). However, the function of short chain fatty acids in renal anemia remains unclear, particularly in non-dialysis patients with early and mid-stage CKD. An integrated study examining both fecal and plasma SCFA levels is lacking. Therefore, our study aimed to explore and compare the fecal and plasma SCFA levels in non-dialysis renal anemia patients and healthy controls to reveal the potential interactions among SCFAs, gut microbiota, and clinical features of renal anemia, which may provide new light on further studies of renal anemia.

## 2 Materials and methods

### 2.1 Standard protocol approvals and patient consents

This study was approved by the Ethics Committee of the First Affiliated Hospital of Zhengzhou University (No. 2018-KY-36). Written informed consent was obtained from all participants. The study was performed in accordance with the STROBE guidelines and relevant ethical regulations.

### 2.2 Participants and sample collection

The patients with renal anemia and healthy controls were recruited from the First Affiliated Hospital of Zhengzhou University. At the time of study design and participant recruitment, renal anemia was diagnosed according to the 2004 European Best Practice Guidelines Working Group (Locatelli et al., 2004) as follows: hemoglobin (Hb) levels were considered to be below normal if they were < 11.5 g/dL in women and < 13.5 g/dL in men (< 12 g/dL in those aged > 70 years), for patients living at altitudes <1,500 m. Current KDIGO guidance defines anemia in adults with CKD as Hb < 12.0 g/dL in women and <13.0 g/dL in men (Tonelli et al., 2026). All patients enrolled in the present study had Hb < 10.0 g/dL and therefore met both the criteria applied at the time of study recruitment and the current definition of anemia.

In the context of a confirmed diagnosis of CKD, renal anemia is diagnosed after comprehensive evaluation and exclusion of other identifiable causes of anemia. In this study, Hb < 10.0 g/dL was predefined as a study-specific inclusion criterion threshold to recruit patients with clinically evident renal anemia and should not be interpreted as the diagnostic threshold for renal anemia.

Controls were accompanying friends or family members of the patients with renal anemia and had no known kidney or urinary system disease. A total of 30 non-dialysis renal anemia patients with Hb < 10.0 g/dL and 20 healthy controls were enrolled. The inclusion criteria were as follows: (1) voluntary agreement to participate in the study; (2) aged ≥ 18 years of age; (3) diagnosed with renal anemia and Hb < 10.0 g/dL; (4) no history of hemodialysis or peritoneal dialysis; The exclusion criteria were as follows: (1) diabetes mellitus, diabetic nephropathy, hypertensive nephropathy or hypertension-related renal damage, lupus nephritis, or other secondary kidney diseases, which were excluded to minimize confounding effects from metabolic disorders, immune-mediated renal injury, and systemic inflammation on gut microbiota, SCFA metabolism, and anemia-related parameters; (2) had a history of inflammatory bowel disease, irritable bowel syndrome, colitis and colon cancer; (3) use of antibiotics or probiotic supplements within 3 months of enrollment;(4) vegetarians.

Fecal and plasm samples, clinical data, and demographic information were collected. Venous blood and fresh feces from each participant were collected. Blood was sampled in the morning after subjects had fasted for at least 12 h. Serum-separating tubes were prepared for further analysis. Fecal samples were collected and transported to the biobank within 2 h for storage at −80 °C until analysis.

### 2.3 DNA extraction and 16S rRNA gene amplification sequencing

The sample’s genomic DNA was extracted using the CTAB procedure. The purity and quantity of the DNA were evaluated on 1% agarose gels. Based on the measured concentration, the DNA was further diluted to a final concentration of 1 ng/μL in sterile water. To target the V3-V4 regions of the 16S rRNA gene, a custom primer set (515F-806R) that included a barcode was used in the PCR amplification process. Each PCR reaction was prepared in a volume of 15 μL, incorporating the Phusion® High-Fidelity PCR Master Mix (New England Biolabs), 2 μM of each primer, and approximately 10 ng of the template DNA. The PCR thermal cycling routine involved an initial denaturation step at 98 °C for 1 min, followed by 30 cycles of denaturation, annealing, and extension at the temperatures of 98 °C, 50 °C, and 72 °C, respectively, for durations of 10 s, 30 s, and 30 s. A final extension period at 72 °C for 5 min was also included. The PCR products were combined with an equal volume of 1X loading buffer containing SYB green, and the mixture was then subjected to gel electrophoresis on a 2% agarose gel to confirm their presence and size. To achieve equal concentrations, the PCR products were mixed at equimolar ratios. Subsequently, these mixed products were purified using the Qiagen Gel Extraction Kit (Qiagen, Germany). For sequencing, libraries were constructed using the TruSeq® DNA PCR-Free Sample Preparation Kit (Illumina) according to the manufacturer’s specifications, incorporating index sequences to distinguish between samples. The quality of the libraries was evaluated using the Qubit@ 2.0 Fluorometer (Thermo Scientific) and the Agilent Bioanalyzer 2,100 system. The libraries were sequenced on the Illumina NovaSeq platform, resulting in 250 bp paired-end reads. The reads were assigned to their respective samples based on barcode information and had their barcode and primer sequences trimmed off. The trimmed reads were then merged using the FLASH tool (V1.2.7) to create spliced sequences, also known as raw tags. The raw tags were subjected to quality filtering following the QIIME (V1.9.1) protocol, which resulted in the generation of high-quality, clean tags. These tags were compared against the Silva database to identify and exclude any chimeric sequences. After removing chimeras, the remaining tags were considered effective tags, ready for further analysis.

### 2.4 Measurement of fecal and plasma SCFA levels

Samples of Methyl tert-butyl ether (MTBE) were acquired from CNW (CNW Technologies, Germany), while MilliQ water was obtained from Millipore, Bradford, USA. For the study, the standards were procured either from CNW (Beijing) or Aladdin (Shanghai). These standards were initially prepared as stock solutions with a concentration of 1 mg/mL in MTBE, which were then kept frozen at −20 °C. These stock solutions were diluted in MTBE to create working concentrations prior to their use in the analysis. To extract SCFAs from fecal samples, 20 mg feces were precisely weighed and placed in a 2 mL EP tube. The tube then received 1 mL of 0.5% phosphoric acid (v/v) and a mini steel ball. The contents were macerated for 10 s, three times, followed by vortexed for 10 min and ultrasonic treatment for 5 min. After centrifugation at 12,000 rpm for 10 min at 4 °C, the supernatant was transferred to a 1.5 mL centrifugal tube containing 0.5 mL MTBE with an internal standard, mixed, and then re-ultrasonicated and centrifuged under the same conditions. Samples of plasma or serum were thawed and vortexed for 1 min prior to analysis. For this, 50 μL were transferred to a 1.5 mL EP tube, followed by the addition of 100 μL of phosphoric acid (0.5% v/v) solution, vortexing for 3 min, and then including 150 μL of MTBE with an internal standard, vortexing again for 3 min and sonifying for 5 min before centrifuging. Then the mixture was centrifuged at 12000 r/min for 10 min at a temperature of 4 °C. The resultant supernatant was then used for gas chromatography tandem mass spectrometry (GC–MS/MS) analysis. The analysis was conducted using an Agilent 7890B gas chromatograph linked to a 7000D mass spectrometer, which was fitted with a DB-FFAP column (30 meters × 0.25 mm i.d. × 0.25 μm film thickness, J&W Scientific, USA). Helium was the carrier gas at a rate of 1.2 mL/min, with the injection performed in split mode and at a volume of 2 μL. The oven temperature program began at 90 °C and escalated through specified rates up to 200 °C, with a hold at each temperature before the end of the run. Samples were analyzed in the multiple reaction monitoring (MRM) mode, with the injector and transfer line temperatures set at 200 °C and 230 °C, respectively.

### 2.5 Statistical analysis

Continuous (quantitative) data are tested for normality using the Shapiro–Wilk test. If the data follow a normal distribution, they are represented using mean ± standard deviation, and the comparison between two groups is performed using an independent samples t-test. If the data do not follow a normal distribution, they are represented using the median (25th percentile, 75th percentile), and the comparison between two groups is performed using the Wilcoxon test. Categorical (qualitative) data are described using frequency (percentage), and the comparison between groups is performed using the *χ*^2^ test or Fisher’s exact test. To control confounding effects of renal function on the results, we performed a 1:1 propensity score matching (PSM) between the mildly anemic group and the moderately anemic group. Matching variables included serum creatinine (SCr) and 24-h urinary protein (24 hUP) excretion, with a caliper set to 0.2 times the standard deviation of the propensity score. The relationships between SCFAs and clinical parameters and between SCFAs and gut microbiota were analyzed by Spearman’s correlation coefficient. We used the multivariate linear regression analysis to explore the correlations between anti-renal anemia medications and the levels of butyric acid. The fecal and plasma levels of butyric acid were dependent factors. Independent variables included the age, sex, and the usage of anti-renal anemia medications. Statistical analyses were performed using R software (version 4.0.3). A two-tailed *p*-value less than 0.05 is considered statistically significant.

## 3 Results

### 3.1 Renal anemia patients displayed altered microbiota composition compared to healthy subjects

**Figure 1:**
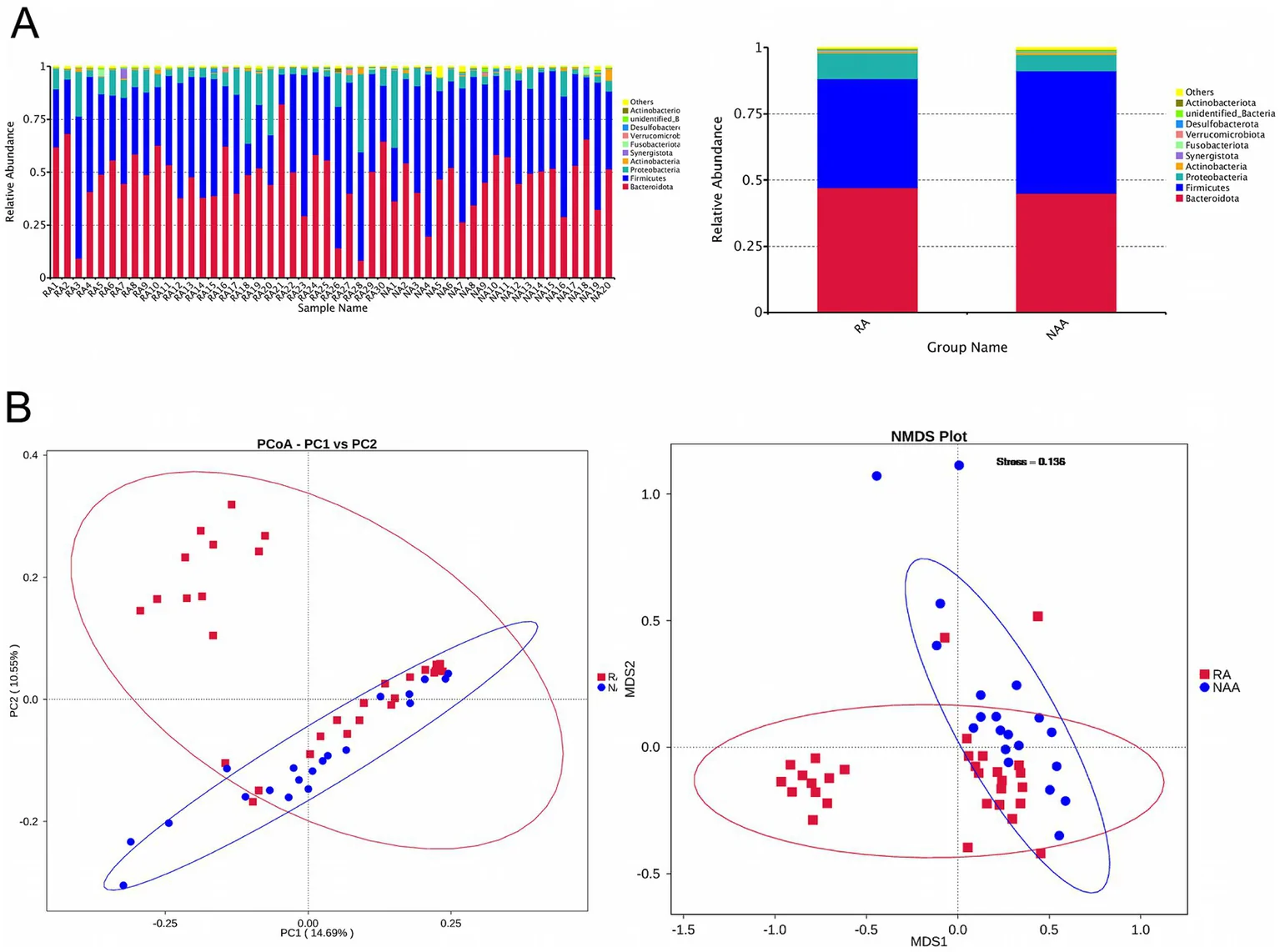
The relative abundance of bacterial and the beta-diversity analysis in renal anemia patients and healthy controls. **(A)** The top 10 bacterial in phylum were selected to generate a bar-cumulative plot of relative abundance. **(B)** PCoA plots and NMDS of bacterial β-diversity based on the unweighted UniFrac distances and Bray–Curtis dissimilarity analyzed according to health status. Patients with renal anemia and controls are colored in red and blue, respectively. RA, Renal anemia; NAA, Healthy controls.

**Figure 2:**
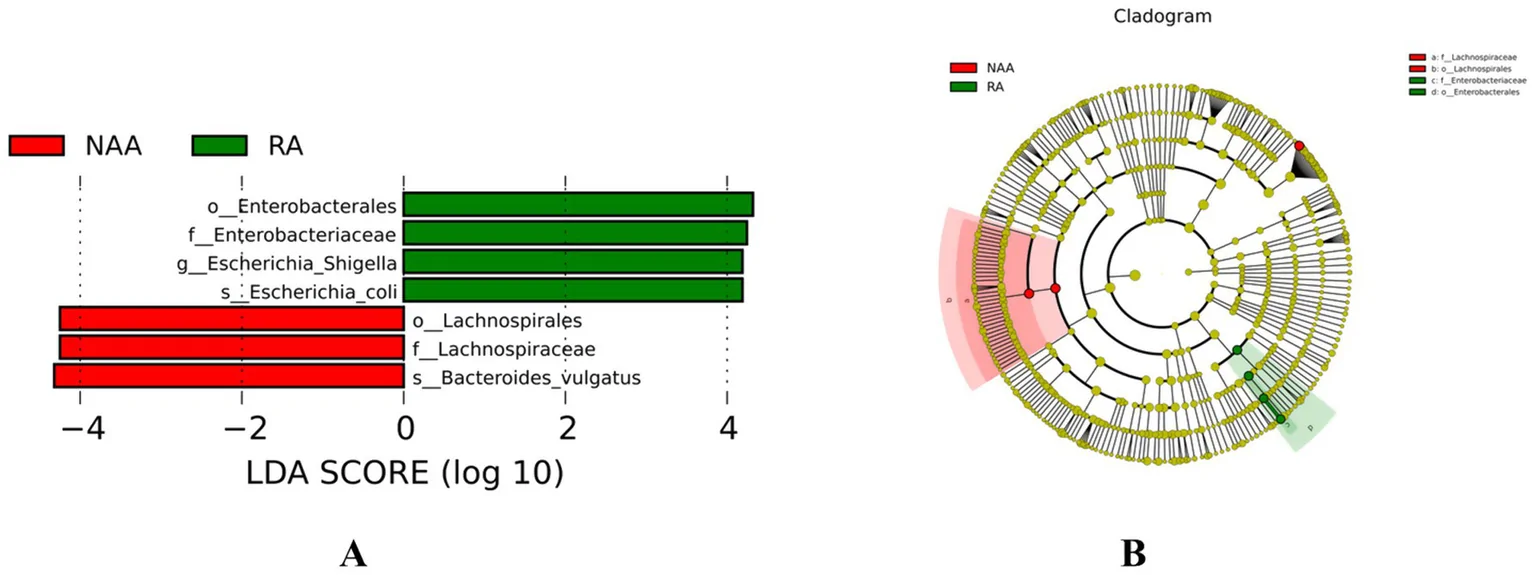
Taxonomic differences of gut microbiota in renal anemia and control groups. **(A)** A linear discriminant analysis (LDA) effect size (LEfSe) analysis revealed significant bacterial differences in gut microbiota between the renal anemia (positive score) and control groups (negative score). LDA scores (log10) > 4 and *p* < 0.05 are shown. **(B)** Cladogram using the LEfSe method showed the differences in enriched taxa in renal anemia (green) versus enriched taxa in controls (red).

Table 1 presents the Characteristics of 30 patients with renal anemia and 20 healthy individuals. Our study analyzed the gut bacterial compositions of this two groups across various taxonomic levels, revealing the Bacteroidetes, Firmicutes, Proteobacteria, and Actinobacteria were the four most abundant phyla in both (Figure 1A). Alpha-diversity was evaluated using Chao1 and ACE indices to estimate microbial richness, and Shannon and Simpson indices to assess microbial diversity and evenness. Compared with healthy controls, renal anemia patients showed a tendency toward higher alpha-diversity; however, the differences were not statistically significant for Chao1 (*p* = 0.512), Shannon index (*p* = 0.599), Simpson index (*p* = 0.364), or ACE (*p* = 0.595). To assess the differences in bacterial composition at the community-level (beta-diversity), we used the Unweighted Unifrac distances and the Bray–Curtis dissimilarity. The principal coordinates analysis (PCoA) and Non-Metric Multi-Dimensional Scaling (NMDS) indicated that the gut microbiota of patients with renal anemia was significantly different from those of the controls (*p* < 0.005 by a Permutational MANOVA (PERMANOVA) implementation using Unweighted Uni-Frac distances and Bray–Curtis dissimilarity, Figure 1B). The outcomes demonstrated that the diversity of gut microbiota is significantly distinct between renal anemia patients and healthy controls. A supervised comparison of the microbiota between renal anemia patients and control groups was performed by linear discriminant analysis (LDA) effect size (LEfSe) analysis without any adjustments, which is commonly used to evaluate the presence and effect size of region-specific OTUs among different groups. We used a logarithmic LDA score cutoff of 4.0 to identify significant taxonomic differences between the renal anemia patients and control groups and found a remarkable difference in fecal microbiota between the renal anemia patients and control groups based on LDA LEfSe analysis (Figure 2). The outcome showed that the relative abundance of Enterobacterales, Enterobacteriaceae, *Escherichia-Shigella*, and *Escherichia-coli* were higher in patients with renal anemia, and the Lachnospiraceae family was decreased in renal anemia patients, which is related to anti-inflammatory effects. In contrast, the relative abundance of Lachnospirales, Lachnospiraceae, and *Bacteroides-vulgatus* was higher in the control group than in the patients with renal anemia. These findings suggested that there were notable differences in the microbiota composition between the two groups.

**Table 1:** Clinical characteristics and SCFA levels in all participants.

Variable	Healthy controls (*n* = 20)	Patients with RA (*n* = 30)	*p*-value
Age, y	49.2 ± 13.84	46.5 ± 12.9	0.485
Sex, *n*			1
Male	10 (50%)	15 (50%)	
Female	10 (50%)	15 (50%)	
Height, cm	167.5 (165, 173.5)	168 (163, 172.75)	0.605
Weight, Kg	63.8 ± 5.75	64.4 ± 5.85	0.722
BMI, kg/m^2^	22.37 ± 1.45	22.77 ± 1	0.3
Hb, g/L	142.12 (133.75, 151)	89.5 (85, 93)	<0.001***
Scr, μmol/L	69.79 (61.75, 76)	328.75 (203.75, 499)	<0.001***
eGFR, ml/min/1.73 m^2^	100.84 (92.1, 111.91)	15.22 (9.28, 28.17)	<0.001***
Urea nitrogen mmol/L	4.99 (4.36, 5.6)	19.73 (14.1, 26.15)	<0.001***
Uric acid, μmol/L	302.06 (275.25, 345.25)	395.5 (354.25, 530.5)	<0.001***
MCV, fl	92.98 ± 3.54	88.91 ± 4.34	0.001***
MCH, pg	30.81 ± 1.19	29.39 ± 2.05	0.003**
MCHC, g/L	332.24 (324.75, 336)	334.5 (322, 338)	0.953
Hct, L/L	0.42 (0.39, 0.45)	0.28 (0.26, 0.29)	<0.001***
RDW, %	12.85 ± 0.62	13.57 ± 1.37	0.015**
RBC, 10^12/L	4.74 (4.42, 4.88)	3.13 (2.93, 3.32)	<0.001***
WBC, 10^9/L	6.5 ± 1.8	6.99 ± 2.36	0.433
PLT, 10^9/L	236 ± 38.87	231.27 ± 74.05	0.77
Percentage of neutrophils, %	55.44 ± 8.4	69.14 ± 11.89	<0.001***
Percentage of lymphocytes, %	36.42 ± 8.93	21.67 ± 10.14	<0.001***
Albumin, g/L	46.75 (45.12, 47.65)	36.5 (32.85, 40.8)	<0.001***
Total bilirubin, μmol/L	9.21 (8.05, 10.62)	3.84 (3.04, 5.16)	<0.001***
TCHO, mmol/L	4.86 ± 0.69	4.1 ± 1.19	0.013*
TG, mmol/L	1.69 (0.79, 2.23)	1.76 (1.01, 2.03)	0.357
LDL, mmol/L	2.88 (2.61, 3.08)	2.36 (1.69, 2.97)	0.024*
HDL, mmol/L	1.42 (1.26, 1.62)	1 (0.86, 1.12)	<0.001***
Fecal levels of SCFAs, mg/g			
Acetic acid	1.73 ± 0.92	2.24 ± 0.86	0.049*
Propionic acid	0.87 (0.49, 1.17)	1.1 (0.62, 1.63)	0.332
Isobutyric acid	0.06 (0.03, 0.08)	0.08 (0.02, 0.11)	0.628
Butyric acid	0.5 (0.34, 0.65)	0.6 (0.39, 0.95)	0.185
Isovaleric acid	0.04 (0.02, 0.07)	0.06 (0.01, 0.1)	0.6
Valeric acid	0.07 (0.01, 0.1)	0.04 (0.01, 0.14)	0.961
Caproic acid	0 (0, 0.01)	0 (0, 0.01)	0.592
Plasma levels of SCFAs, μg/mL			
Acetic acid	0.24 (0.19, 0.39)	0.35 (0.27, 0.47)	0.027*
Propionic acid	0.08 (0.06, 0.1)	0.09 (0.07, 0.12)	0.178
Isobutyric acid	0.02 (0.02, 0.03)	0.02 (0.02, 0.02)	0.002**
Butyric acid	0.02 (0.01, 0.05)	0.02 (0.02, 0.04)	0.44
Isovaleric acid	0.04 (0.03, 0.05)	0.03 (0.02, 0.04)	0.055
Valeric acid	0.01 (0.01, 0.02)	0.01 (0.01, 0.01)	0.5
Caproic acid	0.05 (0.04, 0.05)	0.04 (0.04, 0.05)	0.035*
Anti-RA medications			
HIF-PHIs	/	7 (23.3%)	
Fe+ESAs	/	13 (43.3%)	
Other	/	10 (33.3%)	

RA, renal anemia; BMI, body mass index; Hb, hemoglobin; Scr, serum creatinine; eGFR, estimated glomerular filtration rate; MCV, mean corpuscular volume; MCH, mean corpuscular hemoglobin; MCHC, mean corpuscular hemoglobin concentration; HCT, hematocrit; RDW, red cell distribution width; RBC, red blood cell count; WBC, white blood cell count; PLT, platelet count; TCHO, total cholesterol; TG, triglycerides; LDL, low density lipoprotein; HDL, high density lipoprotein; Other, iron supplementation alone or ESAs alone.

*p < 0.05; **p < 0.01;***p < 0.001.

### 3.2 Fecal and plasma levels of individual SCFAs in patients with renal anemia and controls

**Figure 3:**
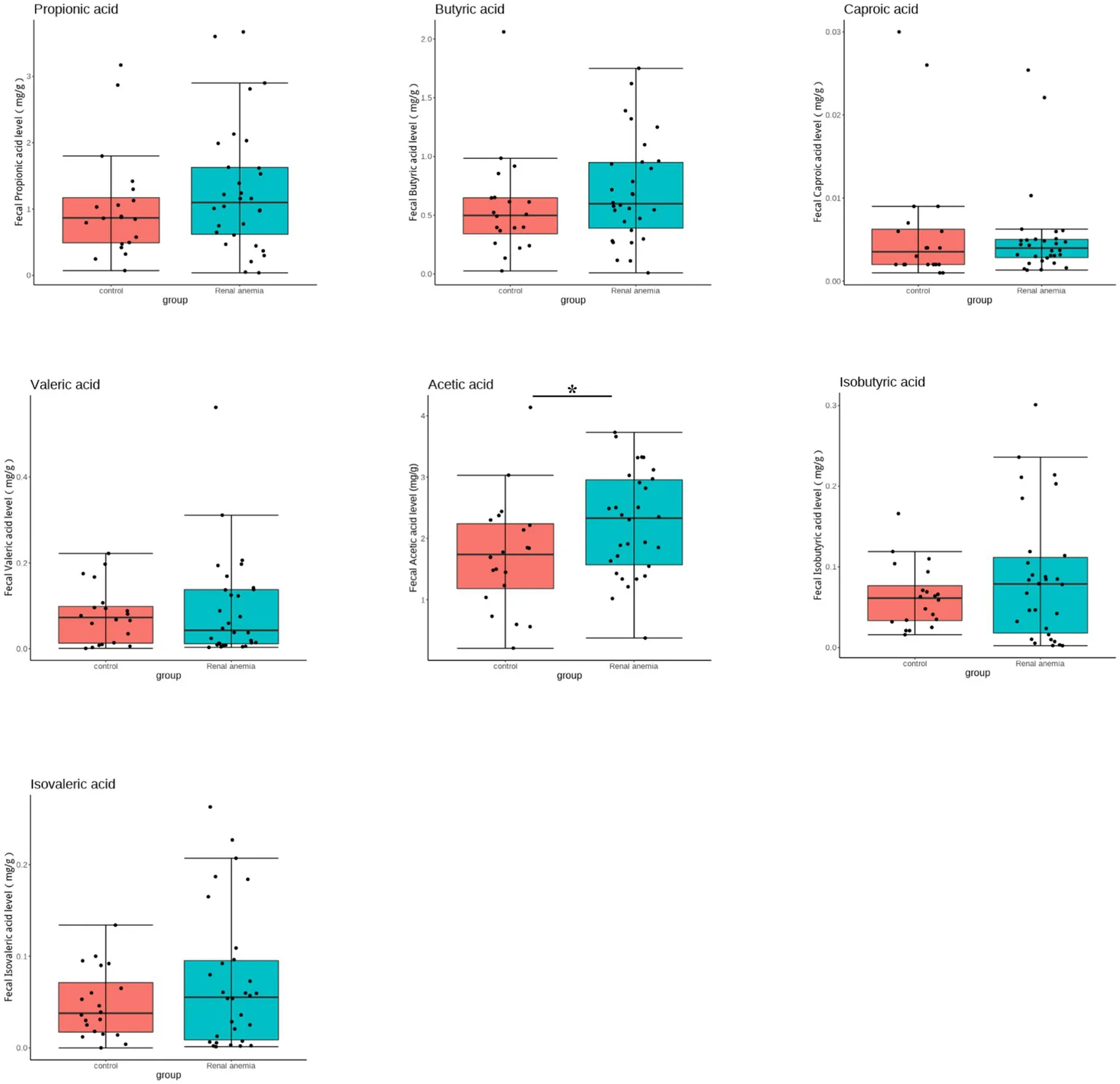
Comparison of fecal levels of short chain fatty acids in patients with renal anemia and healthy controls. Patients with renal anemia and controls are colored in blue and red, respectively. *p < 0.05.

**Figure 4:**
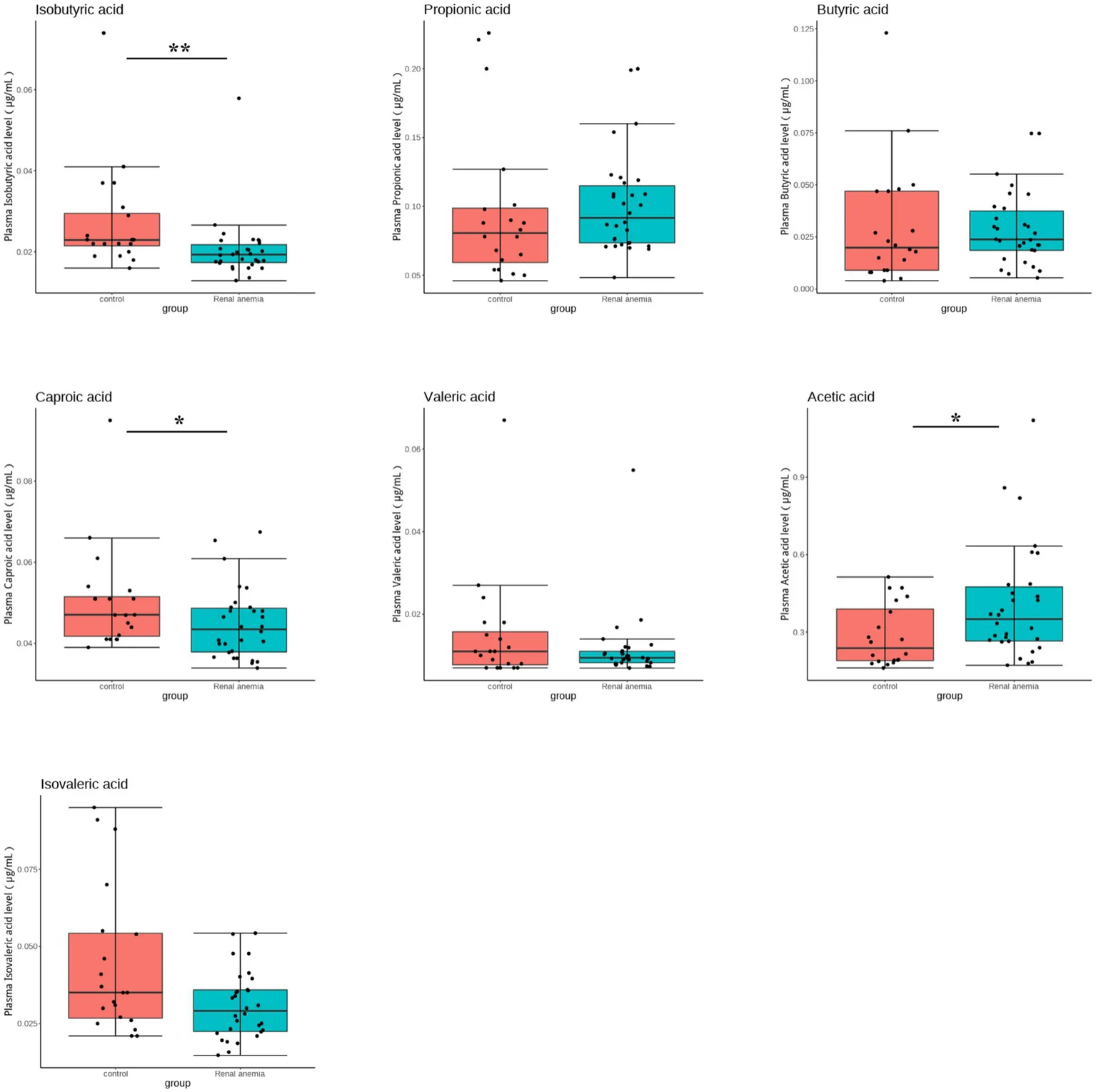
Comparison of plasma levels of short chain fatty acids in patients with renal anemia and healthy controls. Patients with renal anemia and controls are colored in blue and red, respectively. *p < 0.05, **p < 0.01.

The results showed that acetic acid, propionic acid and butyric acid were major components of the SCFAs in fecal and plasma levels. In the comparison of fecal concentrations of SCFAs, patients with renal anemia had significantly higher fecal concentrations of acetic acid (*p* = 0.049) than controls. There were no differences between the two groups in propionic acid (*p* = 0.332), butyric acid (*p* = 0.185), and other types of SCFAs (Table 1 and Figure 3). Similarly, in the comparison of plasma concentrations of SCFAs, acetic acid tended to be also significantly higher in patients with renal anemia (*p* = 0.027). In contrast, plasma concentrations of isobutyric acid (*p* = 0.002) and caproic acid (*p* = 0.035) were increased in controls compared to renal anemia patients (Table 1 and Figure 4).

To further explore whether SCFAs levels differed according to anemia severity, patients with renal anemia were divided into mildly anemic (Hb 90–100 g/L) and moderately anemic (Hb 60–90 g/L) groups based on hemoglobin levels. Before propensity score matching (PSM), fecal isobutyric acid (*p* = 0.019) and isovaleric acid (*p* = 0.028) levels were significantly higher in moderately anemic patients than in mildly anemic patients. No significant differences in plasma SCFA levels were observed between the two subgroups.

To reduce the potential confounding effect of renal function-related variables, 1:1 propensity score matching (PSM) was performed between the mildly anemic group and the moderately anemic group. Matching variables included serum creatinine (SCr) and 24-h urinary protein (24 hUP) excretion, with a caliper set to 0.2 times the standard deviation of the propensity score. After matching, 10 mildly anemic and 10 moderately anemic patients were retained. The standardized mean differences (SMD) for serum creatinine and 24-h urinary protein excretion were 0.116 and 0.084, respectively, indicating that renal function parameters achieved statistical balance between the two groups, supporting the reliability of subsequent analyses investigating the association between anemia severity and SCFAs (Table 2).

**Table 2:** The difference of fecal and plasma levels of SCFAs between mildly anemic and moderately anemic patients before and after propensity-score matching (PSM).

Before PSM				After PSM		
Variable	Mildly anemic patients (*n* = 15)	Moderately anemic patients (*n* = 15)	*p*-value	Mildly anemic patients (*n* = 10)	Moderately anemic patients (*n* = 10)	*p*-value
Age, y	47.6 ± 12.83	45.4 ± 13.32	0.649	44.9 ± 14.53	47.8 ± 13.63	0.651
Sex, *n*			1			1
Male	7 (46.67%)	8 (53.33%)		4 (40%)	4 (40%)	
Female	8 (53.33%)	7 (46.67%)		6 (60%)	6 (60%)	
Hb, g/L	94.73 ± 3.84	82.4 ± 6.49	<0.001***	94.4 ± 4.12	81.2 ± 6.92	<0.001***
Scr, μmol/L	357.5 (205.5, 498)	299 (213.75, 562.5)	0.756	328.75 (210.3, 471.5)	251.75 (207.62, 398.75)	0.650
eGFR, ml/min/1.73 m^2^	14.65 (10.2, 28.08)	15.79 (8.74, 26.65)	0.756	19.04 ± 10.49	20.69 ± 11.26	0.739
24hUP, g	1.86 (0.72, 2.53)	2.58 (1.48, 4.51)	0.110	2.19 (0.85, 2.67)	2.58 (0.91, 3.79)	0.762
Fecal levels of SCFAs, mg/g						
Acetic acid	2.32 ± 0.89	2.17 ± 0.84	0.654	2.02 ± 0.86	2.23 ± 0.94	0.613
Propionic acid	1.24 (0.46, 1.81)	1.01 (0.7, 1.42)	0.803	1.11 ± 0.91	1.37 ± 1.15	0.575
Isobutyric acid	0.02 (0.01, 0.08)	0.09 (0.06, 0.16)	0.019*	0.04 (0.01, 0.08)	0.11 (0.05, 0.18)	0.049*
Butyric acid	0.83 ± 0.47	0.57 ± 0.39	0.115	0.76 ± 0.49	0.49 ± 0.28	0.155
Isovaleric acid	0.01 (0, 0.06)	0.08 (0.04, 0.15)	0.028*	0.02 (0.01, 0.06)	0.09 (0.05, 0.17)	0.034*
Valeric acid	0.02 (0.01, 0.07)	0.12 (0.02, 0.18)	0.130	0.02 (0.01, 0.05)	0.1 (0.03, 0.18)	0.131
Caproic acid	0 (0, 0.01)	0 (0.0)	0.120	0 (0.0.01)	0 (0.0)	0.049*
Plasma levels of SCFAs, μg/mL						
Acetic acid	0.37 (0.27, 0.55)	0.32 (0.25, 0.43)	0.455	0.39 (0.27, 0.58)	0.34 (0.25, 0.47)	0.762
Propionic acid	0.1 (0.07, 0.12)	0.09 (0.07, 0.11)	0.678	0.09 (0.07, 0.12)	0.09 (0.08, 0.11)	0.940
Isobutyric acid	0.02 (0.02, 0.02)	0.02 (0.02, 0.02)	0.419	0.02 (0.02, 0.02)	0.02 (0.02, 0.02)	0.326
Butyric acid	0.02 (0.02, 0.03)	0.02 (0.02, 0.04)	0.756	0.03 (0.02, 0.04)	0.02 (0.02, 0.03)	0.325
Isovaleric acid	0.03 ± 0.01	0.03 ± 0.01	0.750	0.03 ± 0.01	0.03 ± 0.01	0.737
Valeric acid	0.01 (0.01, 0.01)	0.01 (0.01, 0.01)	0.319	0.01 (0.01, 0.01)	0.01 (0.01, 0.01)	1
Caproic acid	0.04 (0.04, 0.05)	0.04 (0.04, 0.05)	0.901	0.05 ± 0.01	0.04 ± 0.01	0.728

*p < 0.05; **p < 0.01; ***p < 0.001.

After PSM, fecal isobutyric acid (*p* = 0.049) and isovaleric acid (*p* = 0.034) levels remained significantly higher in moderately anemic patients than in mildly anemic patients. Fecal caproic acid also differed significantly between the two subgroups after matching (*p* = 0.049). In contrast, plasma SCFA levels did not differ significantly between mildly and moderately anemic patients after PSM.

To further address potential residual confounding by renal function, covariate-adjusted regression was performed in the matched cochort with additional adjustment for SCr, eGFR, and 24-h urine protein. The association between anemia severity and Isobutyric acid remained significant (β = 0.063, *p* = 0.048). These findings suggest that, the severity of anemia may be associated with alterations in specific SCFAs, particularly fecal isobutyric acid. However, because the matched subgroup sample size was small, these results should be interpreted as exploratory and require validation in larger cohorts.

### 3.3 Correlations between fecal and plasma levels of individual SCFAs and clinical characteristics in patients with renal anemia

**Figure 5:**
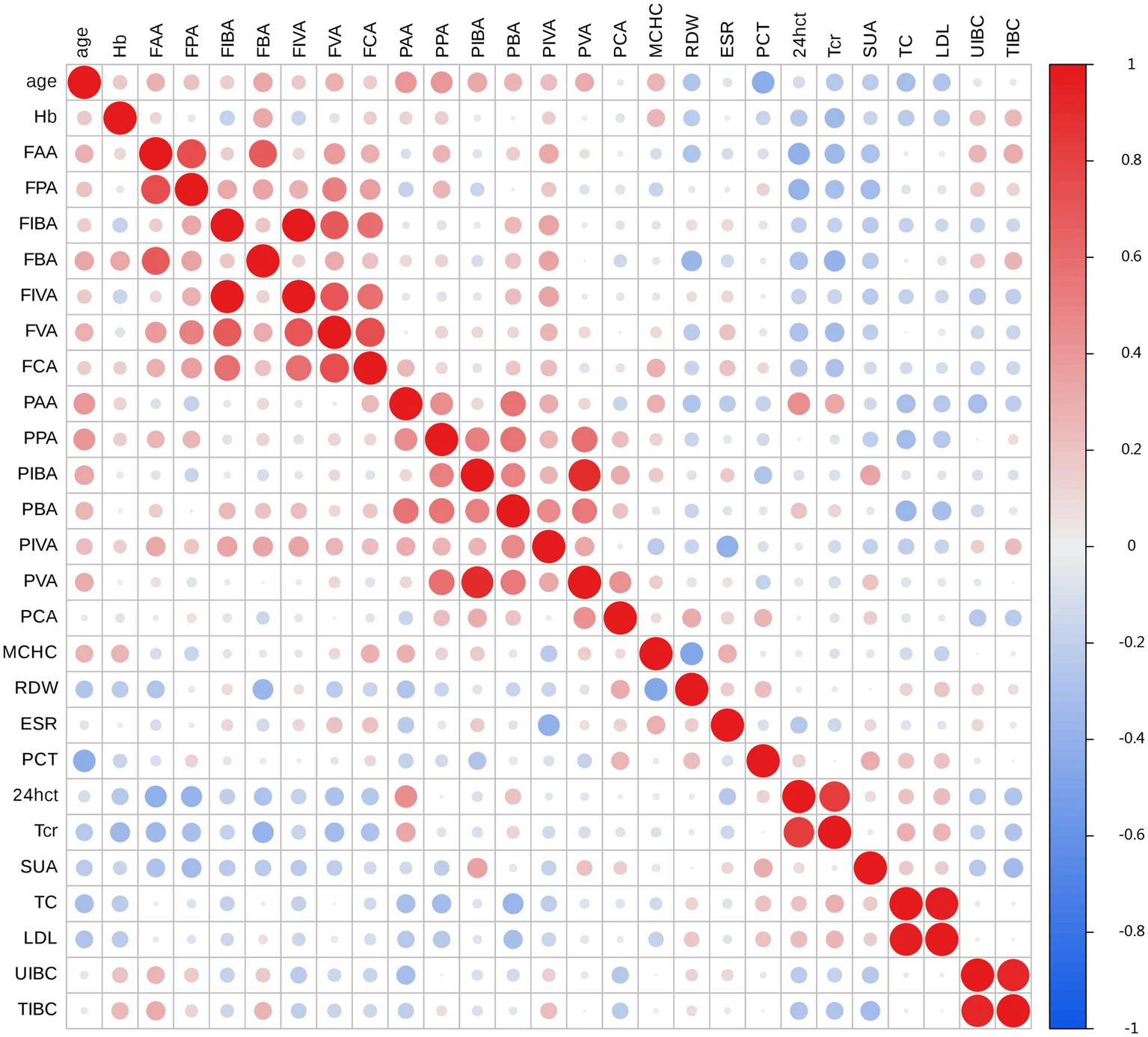
The correlation heatmap of fecal and plasma SCFAs and clinical parameters. The correlation coefficient *r* values are represented by gradient colors, where red and blue cells indicate positive and negative correlations, respectively. FAA, FPA, FIBA, FBA, FIVA, FVA, FCA, fecal levels of acetic acid, propionic acid, isobutyric acid, butyric acid, isovaleric acid, valeric acid, caproic acid; PAA, PPA, PIBA, PBA, PIVA, PVA, PCA, plasma levels of acetic acid, propionic acid, isobutyric acid, butyric acid, isovaleric acid, valeric acid, caproic acid; ESR, erythrocyte sedimentation rate; PCT, procalcitonin; 24hct, 24-h urine protein; Tcr, spot urine protein.

The correlation analyses revealed that both fecal and plasma SCFA levels correlated with clinical features (Table 3 and Figure 5). Specifically, fecal levels of acetic acid (*r* = −0.451, *p* = 0.011; *r* = −0.438, *p* = 0.015) and propionic acid (*r* = −0.405, *p* = 0.026; *r* = −0.361, *p* = 0.05) were negatively correlated with 24-h urine protein (24hUP) and spot urine protein. What’s more, the fecal levels of propionic acid (*r* = −0.383, *p* = 0.037) were negatively correlated with serum uric acid (UA), and the two were also negatively correlated in plasma (*r* = −0.387, *p* = 0.034). Furthermore, the plasma levels of butyric acid were found to be negatively correlated with total cholesterol (TCHO) and low-density lipoprotein (LDL). And the plasma levels of acetic acid (*r* = 0.527, *p* = 0.003) and propionic acid (*r* = 0.467, *p* = 0.009) were positively correlated with age. All of these are consisted with the previous studies. At the same time, the results also showed associations between SCFA levels and several inflammation indicators. For example, plasma isobutyric acid was negatively correlated with procalcitonin (PCT) (*r* = −0.401, *p* = 0.028), and plasma isovaleric acid was negatively correlated with erythrocyte sedimentation rate (ESR) (*r* = −0.376, *p* = 0.04). In the additional analysis, CRP was not significantly correlated with any individual fecal or plasma SCFA. However, inverse trends were observed between CRP and plasma acetic acid (*r* = −0.360, *p* = 0.051), propionic acid (*r* = −0.346, *p* = 0.061), and isovaleric acid (*r* = −0.318, *p* = 0.086) (Table 3 and Supplementary Table 1). Of note, the fecal levels of acetic acid (*r* = 0.37, *p* = 0.044) and caproic acid (*r* = 0.371, *p* = 0.044) were found to be positively correlated with total iron-binding capacity (TIBC) and mean corpuscular hemoglobin concentration (MCHC), respectively. Taken together, these findings suggested that the fecal and plasma levels of individual SCFAs are intimately associated with clinical characteristics. The relevance may be due to the protective effects of SCFAs in host metabolism (den Besten et al., 2013) and potential roles in anemia (Soriano-Lerma et al., 2022), which are in line with previous studies.

**Table 3:** Correlations between fecal and plasma SCFAs and clinical parameters.

Variable	24hUP		Spot urine protein		UA		TIBC		MCHC	
	R	P	R	P	R	P	R	P	R	P
F-AA	−0.46	0.01*	−0.44	0.02*	−0.35	0.06	0.37	0.04*	−0.072	0.71
F-PA	−0.41	0.03*	−0.36	0.05*	−0.38	0.04*	0.10	0.59	−0.09	0.04
F-CA	−0.2	0.29	−0.31	0.09	−0.18	0.34	−0.09	0.62	0.37	0.04*
P-AA	0.28	0.14	0.09	0.65	−0.20	0.29	−0.25	0.19	0.20	0.29
P-PA	−0.01	0.96	−0.14	0.48	−0.39	0.03*	0.04	0.83	0.20	0.30
P-IBA	0.10	0.60	0.01	0.99	0.20	0.30	−0.18	0.35	0.20	0.28
P-BA	0.17	0.37	0.01	0.95	−0.08	0.67	−0.04	0.85	0.18	0.34
P-IVA	−0.01	0.96	−0.11	0.56	−0.27	0.15	0.15	0.43	−0.26	0.17

Variable	Age		TCHO		LDL		PCT		ESR		CRP	
	R	P	R	P	R	P	R	P	R	P	R	P
F-AA	0.35	0.06	−0.06	0.75	−0.04	0.84	−0.02	0.91	−0.10	0.59	−0.07	0.71
F-PA	0.28	0.13	−0.21	0.26	−0.25	0.19	0.15	0.43	−0.06	0.74	0.04	0.84
F-CA	0.11	0.57	−0.20	0.30	−0.20	0.58	0.23	0.22	0.12	0.54	0.03	0.86
P-AA	0.53	0.003**	−0.30	0.11	−0.25	0.19	−0.26	0.16	−0.27	0.15	−0.36	0.051
P-PA	0.47	0.01**	−0.26	0.17	−0.23	0.22	−0.05	0.81	0.03	0.87	−0.35	0.06
P-IBA	0.25	0.18	0.07	0.73	0.06	0.77	−0.40	0.03*	0.10	0.60	−0.20	0.30
P-BA	0.27	0.15	−0.47	0.01**	−0.37	0.04*	0.08	0.70	−0.05	0.81	−0.19	0.31
P-IVA	0.24	0.21	−0.21	0.26	−0.16	0.40	−0.12	0.52	**−**0.38	0.04*	−0.32	0.09

*p < 0.05; **p < 0.01; ***p < 0.001.

### 3.4 The possible influence of anti-renal anemia medications on fecal and plasma SCFAs

To explore the potential influence of anti-anemic medications on SCFA profiles, patients were classified into three groups according to their treatment regimens: HIF-PHIs, ESAs plus iron supplementation, and other treatments (patients receiving iron supplementation alone or ESAs alone). Detailed information on individual anti-anemic medications, including drug names, dosages, administration frequencies, routes, and treatment duration, is provided in Supplementary Table 2. Among the SCFAs examined, fecal butyric acid (*p* = 0.046) and plasma butyric acid (*p* = 0.015) differed significantly among the three treatment groups (Supplementary Table 3). The ESAs plus iron supplementation group showed higher fecal butyric acid levels. Given the small sample size and non-randomized treatment allocation, these findings should be considered exploratory. These findings suggest a possible association between anti-anemic treatment regimens and SCFA profiles, however, further research is still required.

### 3.5 Correlations between gut microbial taxa and fecal or plasma SCFAs

**Figure 6:**
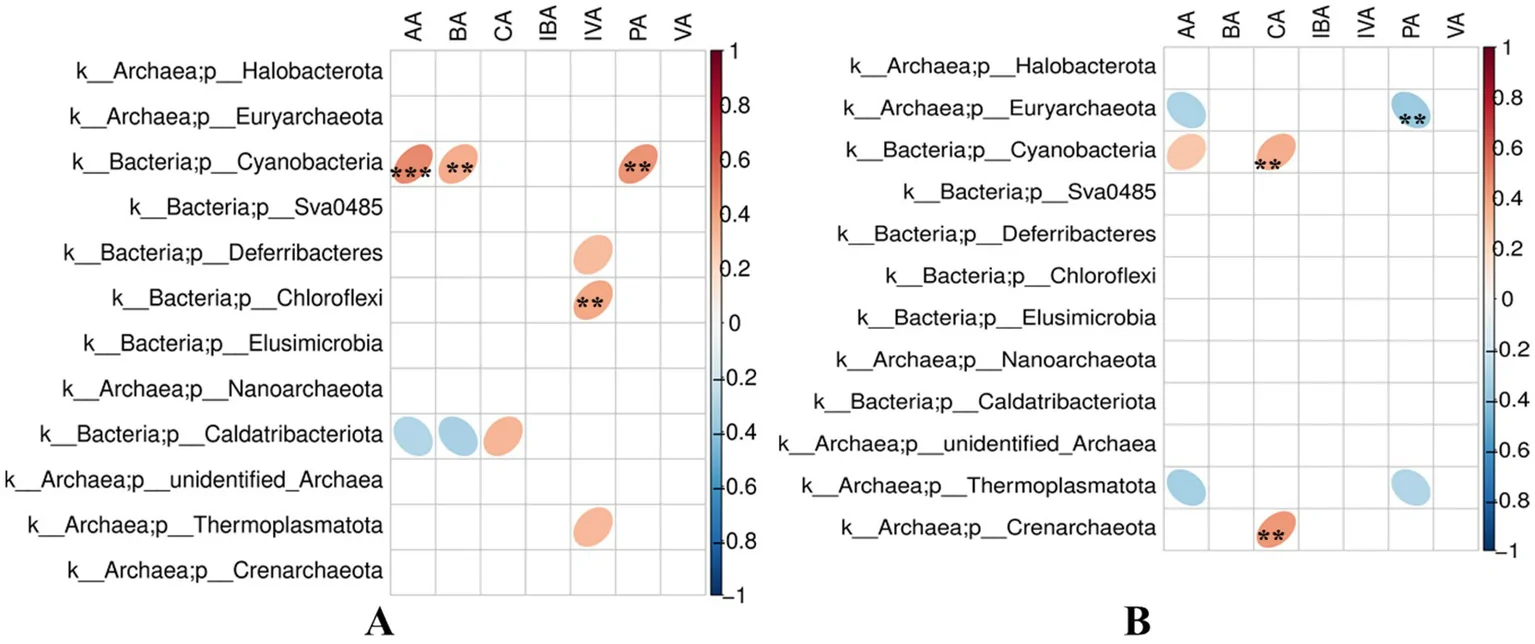
Heat maps representing the Spearman correlation of the relative abundance of different bacteria and the fecal and plasma levels of SCFAs in the phylum level. **(A)** The correlation of the relative abundance of different bacteria and the plasma levels of SCFAs; **(B)** The correlation of the relative abundance of different bacteria and the fecal levels of SCFAs. The *r* values are represented by gradient colors, where red and blue cells indicate positive and negative correlations, respectively. *p < 0.05; **p < 0.01; ***p < 0.001.

We explored the correlations between the relative abundance of gut microbial taxa and fecal or plasma SCFA concentrations using Spearman correlation analysis. At the phylum level, Cyanobacteria was positively correlated with fecal acetic acid and caproic acid, as well as with plasma acetic acid, propionic acid, and butyric acid. The phylum-level correlation results are shown in Figure 6.

To further identify specific bacterial taxa associated with SCFA profiles, we performed genus-level correlation analyses between differentially abundant genera and fecal or plasma SCFA concentrations. Several genus-level taxa within the Lachnospiraceae family were correlated with SCFA levels. For example, f__Lachnospiraceae;g__GCA-900066575 was positively correlated with acetic acid levels in both plasma and feces, and f__Lachnospiraceae;g__Eisenbergiella was positively correlated with plasma propionic acid. In contrast, f__Lachnospiraceae;g__Oribacterium was negatively correlated with fecal isobutyric acid and isovaleric acid. These genus-level correlation patterns are presented in Supplementary Figures S1A,B.

Taken together, these findings suggest that alterations in specific gut microbial taxa, particularly taxa within the Lachnospiraceae family and Cyanobacteria, are associated with fecal and plasma SCFA profiles in patients with renal anemia.

## 4 Discussion

Based on what we have learned currently, this is the first study simultaneously exploring both fecal and plasma levels of various SCFA types and correlating them with alterations in gut microbiota and clinical characteristics in patients with renal anemia, especially in non-dialysis patients with severe anemia and excluding secondary renal disease. We found a distinctive profile of the gut microbial community in patients with renal anemia compared to health controls. What’s more, patients with renal anemia had higher fecal and plasma SCFA concentrations compared to controls, and some types SCFA levels were significantly different between the two groups. We further demonstrated that the fecal and plasma SCFA levels were associated with gut microbiota changes and clinical characteristics of renal anemia. We also found that anemia severity may be associated with specific fecal SCFA alterations.

In our study, 16S rRNA gene sequencing confirmed that the gut microbiota was significantly different between renal anemia patients and healthy controls, which has been extensively demonstrated in previous studies (Zhao et al., 2021). We observed that Enterobacterales (Vaziri et al., 2013), E*scherichia-Shigella* (Zhong et al., 2020; Hu et al., 2020; Ren et al., 2020; Wu et al., 2020; Lun et al., 2019), and *Escherichia-coli* (Wu et al., 2020; De Angelis et al., 2014) were more abundant in renal anemia patients, which is in line with several previous studies on CKD. Similar to the current study’s findings, Ren et al. (Ren et al., 2020) also found that Lachnospiraceae were less abundant in patients with CKD than in healthy controls. Consistently, this outcome was also observed in other studies about CKD (Wu et al., 2020; Lun et al., 2019). Of note, in the phylum level, our data showed that Cyanobacteria was more abundant in renal anemia patients than healthy controls [(0.000139 ± 0.000213) VS (0.000027 ± 0.000048) *t*-tests, *p* = 0.009], which has not been reported in the previous studies on CKD but was found to exert influence on anemia and immune function (Selmi et al., 2011).

We also evaluated the fecal and plasma SCFA levels between renal anemia patients and healthy controls and extensively analyzed the interactions between SCFAs and clinical characteristics of renal anemia. It should be noted that fecal and plasma SCFA profiles may not be fully concordant because they reflect different physiological compartments. Fecal SCFA concentrations mainly represent luminal microbial production and residual unabsorbed SCFAs, whereas plasma SCFA concentrations are influenced by intestinal absorption, portal transport, first-pass hepatic metabolism, peripheral utilization, systemic clearance, and gut permeability. In CKD, intestinal barrier dysfunction and altered gut permeability may further affect the translocation of microbial metabolites, including SCFAs, into the circulation and therefore represents a potential confounding factor when interpreting plasma SCFA concentrations. Because intestinal permeability was not directly assessed in the present study, its contribution to the observed plasma SCFA alterations cannot be determined. These mechanisms may help explain why fecal and plasma SCFA alterations were not identical in the present study (Szrejder and Piwkowska, 2025; Sivaprakasam et al., 2017; Boets et al., 2017).

In stool, the acetic acid was significantly different between the two groups and was negatively correlated with 24-h urine protein (24hUP) and spot urine protein. This is consistent with previous findings confirming the protective effects of acetic acid on the kidney (Li et al., 2020; Kawabata et al., 2023). Similarly, propionic acid was also negatively correlated with 24-h urine protein, spot urine protein, and serum uric acid (SUA). The metabolic and renal protective effects of SCFAs have been widely demonstrated (den Besten et al., 2013; Li et al., 2020; Wang et al., 2019). In plasma, in addition to the negative correlations between propionic acid and SUA, butyric acid was also negatively correlated with TC and LDL. Our results support the previous studies that butyric acid is of great benefit in lipid metabolism (Xu et al., 2021; Coppola et al., 2022). Moreover, we observed that isobutyric acid and isovaleric acid were negatively correlated with PCT and ESR, respectively. A variety of evidence has demonstrated the anti-inflammatory effects of SCFAs (Corrêa-Oliveira et al., 2016). The possible pathways that SCFAs exerting anti-inflammatory effects may include reducing the secretion of pro-inflammatory cytokines and chemokines, inhibiting the activation of NF-κB in the host immune cells by binding to G-protein-coupled receptors (GPRs) 43 and 41 (Säemann et al., 2000; Tedelind et al., 2007), and enhancing the host response to inflammation by epigenetically regulating gene expression through inhibiting histone deacetylases, which can induce the differentiation of colonic Treg cells (Furusawa et al., 2015; Galvão et al., 2018; Vieira et al., 2017). So, our study is consisted with the previous outcomes. Notably, we found that the fecal and plasma acetic acid was correlated with TIBC and UIBC. The fecal caproic acid was also related to MCHC. At the same time, the fecal levels of isobutyric acid and isovaleric acid are significantly higher in moderately anemic groups than in mildly anemic patients. In line with these findings, the potential roles of SCFAs in iron metabolism and anemia have been proved by previous studies (Soriano-Lerma et al., 2022; Zhao et al., 2023; Das et al., 2020; McClorry et al., 2018). SCFAs have been proposed as mediators of iron absorption. Previous studies have revealed an intimate relationship between HIF, iron metabolism, and SCFAs (Liu et al., 2022). In the anemia severity subgroup analysis, we used PSM to reduce the potential confounding effect of renal function-related variables. Despite the limited sample size, PSM analysis suggested that anemia severity may be associated with specific fecal SCFA alterations, particularly fecal isobutyric acid. Future validation in larger cohorts is warranted to confirm this finding. Given the elaborate interactions, we explored the possible influence of anti-renal anemia medications on fecal and plasma SCFAs. The outcomes showed that both the fecal and plasma levels of butyric acid were significantly different among the different types of medication use. These findings indicated a possible association between anti-anemic treatment regimens and SCFA profiles, but more in-depth studies are still needed.

In addition, we revealed the correlations between differentially expressed bacteria and SCFA concentrations in feces or plasma between renal anemia patients and healthy controls. Genera within the Lachnospiraceae family belong to the core of the gut microbiota and are the main producers of SCFAs. Their anti-inflammatory and immunomodulating effects on the human gut have been validated in previous studies (Vacca et al., 2020). In our research, we found a significantly lower abundance of Lachnospiraceae in patients with renal anemia and many genus-level microorganisms belonging to it are positively correlated with some types of SCFAs in plasma. Overall, our data highlight the protective roles of Lachnospiraceae in the organism. Moreover, we found a significantly higher abundance of Cyanobacteria in renal anemia patients and it is positively correlated with many types of SCFAs both in fecal and plasma. Although the possible effect of Cyanobacteria has not been reported in the previous studies on CKD, it was found to play a beneficial role on anemia and immune function (Selmi et al., 2011). Therefore, further mechanistic studies of the Cyanobacteria in renal anemia are needed.

CKD itself is known to profoundly influence the gut microbiota through multiple mechanisms, including uremic toxin accumulation, chronic inflammation, impaired intestinal barrier function, and dietary restrictions (Vaziri et al., 2013). Therefore, the gut microbiota and SCFA alterations observed in our study may not be solely attributable to anemia, but rather reflect the broader impact of CKD-related dysbiosis. Given the markedly reduced eGFR in our patient cohort compared to healthy controls, the current study design cannot fully isolate the independent contribution of anemia. Nevertheless, there is strong mechanistic reasoning to suggest that renal anemia acts as a key amplifying factor that further aggravates CKD-associated microbial imbalance. Reduced hemoglobin levels lead to systemic and intestinal tissue hypoxia, which can fundamentally alter the intestinal ecological niche and selectively affect the growth of specific microbial taxa, particularly anaerobic SCFA-producing bacteria (Liu et al., 2022). Furthermore, disordered iron metabolism, iron supplementation, and impaired erythropoietin responsiveness—all hallmark features of renal anemia—can directly interact with gut microbial composition and metabolism (Jaeggi et al., 2015; Trompette et al., 2014). Thus, while CKD sets the baseline for dysbiosis, concurrent anemia likely modifies and amplifies these disturbances.

However, our results are in partial agreement with those previously produced by others. For example, we identified contradictory results that patients with renal anemia had higher fecal and plasma SCFA concentrations compared to controls, which is contrary to previous studies on CKD (Wang et al., 2019). A similar unexpected increase in fecal SCFAs was reported in patients with iron deficiency anemia (IDA) (Soriano-Lerma et al., 2022), and the researchers assumed that such an increase in SCFAs during the development of IDA suggests that trade-off mechanisms are taking place to compensate for physiological alterations during the disease, namely alterations in the intestinal tract, IDA-derived insulin resistance or appetite reduction. The same may also occur in renal anemia. At the same time, an unexpected increase in plasma SCFAs was also found in patients with Parkinson Disease (PD) (Chen et al., 2022) and patients with infection (salmonellosis) and auto-inflammatory conditions (familial Mediterranean fever [FMF]) of the intestine (Ktsoyan et al., 2016). The two studies attributed elevated SCFAs in plasma to “leaky gut phenomenon”, which means that increased intestinal permeability allows SCFAs to enter the systemic circulation with low-grade inflammation, implying an intestinal barrier malfunction. Similarly, persistent inflammation is frequently observed in CKD patients, and renal anemia is tightly associated with inflammatory status (Radić et al., 2017; Yilmaz et al., 2011). Other possible explanations for this divergence may come from differences in the methodologies and patient enrollment criteria.

This study has several limitations. First, this is a single-center observational study with a relatively small sample size, and no formal prior power calculations were performed. After propensity score matching of patients with mild and moderate anemia, only 10 patients remained in each subgroup; therefore, the results based on propensity score matching should be considered exploratory rather than definitive. Second, the present study did not include an eGFR-matched control group of non-dialysis CKD patients without anemia. Such a group would have provided a more appropriate comparison for separating the influence of CKD itself from the potential contribution of anemia. However, in real-world clinical practice, anemia is common among patients with end-stage CKD, and individuals with markedly reduced kidney function but without anemia are relatively difficult to recruit. Third, dietary fiber intake, bowel habits, constipation status, and acid–base parameters were not systematically assessed, although vegetarians were excluded to reduce the influence of markedly different dietary patterns. These factors may affect SCFA production, absorption, and circulating concentrations, particularly in CKD populations. Finally, although patients with diabetes mellitus, diabetic nephropathy, hypertensive nephropathy or hypertension-related renal damage, lupus nephritis, and other secondary kidney diseases were excluded, residual confounding from inflammatory status, iron metabolism, disease severity, and other CKD-related factors cannot be fully ruled out. Larger multicenter studies with well-matched CKD control groups and comprehensive dietary and clinical assessments are needed to validate these findings.

## 5 Conclusion

Our findings suggest that gut microbiota alterations and specific SCFA profiles are associated with the clinical characteristics of patients with renal anemia in the context of CKD. Moreover, anemia severity may be associated with specific fecal SCFA alterations. These results support further investigation of the gut microbiota–SCFA axis in renal anemia. Larger studies including eGFR-matched non-anemic CKD controls are required to clarify the independent contribution of anemia and the potential biological significance of these associations.

## Data Availability

The datasets presented in this study can be found in online repositories. The names of the repository/repositories and accession number(s) can be found in the article/Supplementary material.
