# Supplementary material for: Analysis of gut microbiota and fecal/plasma short-chain fatty acids in non-dialysis renal anemia patients: associations with clinical parameters
Source: Front Microbiol. 2026 Sep 7;17:1838946. doi: 10.3389/fmicb.2026.1838946 (PMC13591154; doi:10.3389/fmicb.2026.1838946)
Supplement: Supplementary file 1 [file Supplementary_file_1.DOCX]

**Supplementary Table 1. Spearman correlations between CRP and fecal/plasma SCFAs**

| **SCFA** | **Spearman r** | **p value** |
| --- | --- | --- |
| Fecal acetic acid | −0.070 | 0.714 |
| Fecal propionic acid | 0.039 | 0.839 |
| Fecal isobutyric acid | 0.160 | 0.400 |
| Fecal butyric acid | −0.124 | 0.513 |
| Fecal isovaleric acid | 0.155 | 0.413 |
| Fecal valeric acid | −0.150 | 0.429 |
| Fecal caproic acid | 0.033 | 0.864 |
| Plasma acetic acid | −0.360 | 0.051 |
| Plasma propionic acid | −0.346 | 0.061 |
| Plasma isobutyric acid | −0.195 | 0.301 |
| Plasma butyric acid | −0.193 | 0.307 |
| Plasma isovaleric acid | −0.318 | 0.086 |
| Plasma valeric acid | −0.229 | 0.223 |
| Plasma caproic acid | 0.279 | 0.135 |

**Supplementary Table 2. Detailed information on anti-anemic medications administered to patients with renal anemia**

| **Patient ID** | **Anti-anemic medication** | **Dose** | **Frequency** | **Route** | **Duration before sample collection (days)** |
| --- | --- | --- | --- | --- | --- |
| 1 | Ferrous succinate tablets | 0.1 g per dose | Twice daily | Oral | 667 |
|  | Recombinant human erythropoietin (rhEPO) injection | 10,000 IU per dose | Once weekly | Subcutaneous | 667 |
| 2 | Roxadustat capsules | 70 mg per dose | Three times weekly (Mon/Wed/Fri) | Oral | 3 |
| 3 | Erythropoietin injection | 6,000 IU per dose | Twice weekly (Mon/Thu) | Subcutaneous | 318 |
| 4 | Iron polysaccharide complex capsules | 0.15 g (1 capsule) per dose | Once daily | Oral | 44 |
| 5 | Iron polysaccharide complex capsules | 0.15 g (1 capsule) per dose | Once daily | Oral | 81 |
|  | Recombinant human erythropoietin (rhEPO) injection | 10,000 IU per dose | Twice weekly (Mon/Thu) | Subcutaneous | 81 |
| 6 | Iron polysaccharide complex capsules | 0.15 g (1 capsule) per dose | Once nightly | Oral | 3 |
|  | Recombinant human erythropoietin (rhEPO) injection | 6,000 IU per dose | Twice weekly (Mon/Thu) | Subcutaneous | 3 |
| 7 | Roxadustat capsules | 100 mg per dose | Three times weekly (Mon/Wed/Fri) | Oral | 3 |
| 8 | Iron polysaccharide complex | 0.15 g (1 capsule) per dose | Once daily | Oral | 82 |
|  | Recombinant human erythropoietin (rhEPO) injection | 6,000 IU per dose | Once weekly (Tue) | Subcutaneous | 82 |
| 9 | Iron polysaccharide complex capsules | 0.15 g (1 capsule) per dose | Once nightly | Oral | 3 |
|  | Recombinant human erythropoietin (rhEPO) injection | 6,000 IU per dose | Twice weekly (Tue/Fri) | Subcutaneous | 3 |
| 10 | Iron polysaccharide complex | 0.15 g (1 capsule) per dose | Twice daily | Oral | 3 |
|  | Recombinant human erythropoietin (rhEPO) injection | 10,000 IU per dose | Twice weekly | Subcutaneous | 3 |
| 11 | Roxadustat capsules | 100 mg per dose | Three times weekly | Oral | 3 |
| 12 | Ferrous succinate tablets | 0.1 g per dose | Three times daily | Oral | 201 |
|  | Erythropoietin injection | 10,000 IU per dose | Once weekly | Subcutaneous | 201 |
| 13 | Iron polysaccharide complex capsules | 0.15 g (1 capsule) per dose | Once daily | Oral | 3 |
|  | Recombinant human erythropoietin (rhEPO) injection | 6,000 IU per dose | Twice weekly (Mon/Thu) | Subcutaneous | 3 |
| 14 | Iron polysaccharide complex capsules | 0.15 g (1 capsule) per dose | Once nightly | Oral | 3 |
|  | Recombinant human erythropoietin (rhEPO) injection | 10,000 IU per dose | Twice weekly (Tue/Fri) | Subcutaneous | 3 |
| 15 | Roxadustat capsules | 70 mg per dose (20 mg + 50 mg; 1 capsule each) | Three times weekly (Mon/Wed/Fri) | Oral | 3 |
| 16 | Recombinant human erythropoietin (rhEPO) injection | 10,000 IU per dose | Twice weekly (Tue/Fri) | Subcutaneous | 107 |
| 17 | Ferrous succinate tablets | 0.1 g per dose | Three times daily | Oral | 34 |
| 18 | Roxadustat capsules | 100 mg per dose (50 mg × 2 capsules) | Three times weekly; Mon/Wed/Fri also recorded | Oral | 3 |
|  | Iron polysaccharide complex capsules | 0.15 g (1 capsule) per dose | Once nightly | Oral | 3 |
| 19 | Iron polysaccharide complex | 0.15 g (1 capsule) per dose | Once daily | Oral | 3 |
| 20 | Ferrous succinate tablets | 0.1 g per dose | Three times daily | Oral | 13 |
|  | Erythropoietin injection | 10,000 IU per dose | Once weekly | Subcutaneous | 13 |
| 21 | Roxadustat capsules | 100 mg per dose (50 mg × 2 capsules) | Three times weekly (Mon/Wed/Fri) | Oral | 10 |
| 22 | Erythropoietin injection | 6,000 IU per dose | Once weekly | Subcutaneous | 3 |
| 23 | Recombinant human erythropoietin (rhEPO) injection | 10,000 IU per dose | Twice weekly | Subcutaneous | 9 |
|  | Ferrous succinate tablets | 0.1 g per dose | Three times daily | Oral | 9 |
| 24 | Roxadustat capsules | 100 mg per dose (50 mg × 2 capsules) | Three times weekly (Mon/Wed/Fri) | Oral | 3 |
| 25 | Ferrous succinate tablets | 0.1 g per dose | Three times daily | Oral | 3 |
|  | Recombinant human erythropoietin (rhEPO) injection | 10,000 IU per dose | Once weekly | Subcutaneous | 3 |
| 26 | Shengxuebao Mixture | 10 mL per dose | Three times daily | Oral | 3 |
|  | Recombinant human erythropoietin (rhEPO) injection | 6,000 IU per dose | Once weekly | Subcutaneous | 3 |
| 27 | Roxadustat capsules | 100 mg per dose (50 mg × 2 capsules) | Three times weekly (Mon/Wed/Fri) | Oral | 3 |
| 28 | Recombinant human erythropoietin (rhEPO) injection | 10,000 IU per dose | Once weekly | Subcutaneous | 3 |
| 29 | Recombinant human erythropoietin (rhEPO) injection | 10,000 IU per dose | Once weekly | Subcutaneous | 3 |
| 30 | Ferrous succinate tablets | 0.1 g per dose | Twice daily | Oral | 3 |
|  | Erythropoietin injection | 6,000 IU per dose | Twice weekly | Subcutaneous | 3 |

Notes: Treatment duration was defined as the interval between initiation of anti-anemic therapy and fecal/plasma sample collection. IU, international units; rhEPO, recombinant human erythropoietin. For patients receiving more than one anti-anemic medication, each medication is listed on a separate row.

**Supplementary Table 3. Comparison of fecal and plasma SCFA concentrations according to anti-anemic treatment regimen**

| **Variable** | **HIF-PHIs group (n = 7)** | **ESAs + iron group (n = 13)** | **Other treatments (n = 10)** | **Statistic** | **p value** |
| --- | --- | --- | --- | --- | --- |
| Hemoglobin | 84.71 ± 7.89 | 89.92 ± 8.79 | 89.50 ± 7.46 | 1.023 | 0.373 |
| Fecal acetic acid | 2.06 ± 0.83 | 2.44 ± 0.76 | 2.11 ± 1.02 | 0.614 | 0.549 |
| Fecal propionic acid | 0.98 (0.69, 1.39) | 1.22 (0.98, 1.99) | 0.88 (0.32, 1.60) | 1.035 | 0.596 |
| Fecal isobutyric acid | 0.10 (0.02, 0.16) | 0.08 (0.02, 0.12) | 0.07 (0.02, 0.08) | 0.959 | 0.619 |
| Fecal butyric acid | 0.54 ± 0.32 | 0.93 ± 0.45 | 0.53 ± 0.42 | 3.451 | **0.046*** |
| Fecal isovaleric acid | 0.09 (0.01, 0.15) | 0.05 (0.01, 0.11) | 0.06 (0.01, 0.07) | 0.405 | 0.817 |
| Fecal valeric acid | 0.12 (0.01, 0.18) | 0.05 (0.02, 0.14) | 0.03 (0.01, 0.08) | 1.767 | 0.413 |
| Fecal caproic acid | 0 (0, 0.01) | 0 (0, 0.01) | 0 (0, 0) | 5.778 | 0.056 |
| Plasma acetic acid | 0.48 (0.40, 0.55) | 0.28 (0.26, 0.45) | 0.31 (0.28, 0.37) | 2.988 | 0.224 |
| Plasma propionic acid | 0.11 (0.08, 0.15) | 0.09 (0.07, 0.12) | 0.09 (0.07, 0.10) | 0.744 | 0.689 |
| Plasma isobutyric acid | 0.02 (0.02, 0.02) | 0.02 (0.02, 0.02) | 0.02 (0.02, 0.02) | 3.674 | 0.159 |
| Plasma butyric acid | 0.03 (0.02, 0.04) | 0.03 (0.02, 0.05) | 0.02 (0.01, 0.02) | 8.451 | **0.015*** |
| Plasma isovaleric acid | 0.03 ± 0.01 | 0.03 ± 0.01 | 0.03 ± 0.01 | 0.005 | 0.995 |
| Plasma valeric acid | 0.01 (0.01, 0.01) | 0.01 (0.01, 0.01) | 0.01 (0.01, 0.01) | 3.267 | 0.195 |

Notes: Data are presented as mean ± SD for normally distributed variables or median (25th percentile, 75th percentile) for non-normally distributed variables. Group comparisons were performed using one-way ANOVA or the Kruskal-Wallis test, as appropriate. HIF-PHIs group: patients treated with a hypoxia-inducible factor prolyl hydroxylase inhibitor; ESAs + iron group: patients receiving erythropoiesis-stimulating agents plus iron supplementation; Other treatments: patients receiving iron supplementation alone or ESAs alone. *p < 0.05. SCFA, short-chain fatty acid; ESAs, erythropoiesis-stimulating agents; HIF-PHIs, hypoxia-inducible factor-prolyl hydroxylase inhibitors.
